# *SwissGenVar*: A Platform for Clinical-Grade Interpretation of Genetic Variants to Foster Personalized Healthcare in Switzerland

**DOI:** 10.3390/jpm14060648

**Published:** 2024-06-17

**Authors:** Dennis Kraemer, Dillenn Terumalai, Maria Livia Famiglietti, Isabel Filges, Pascal Joset, Samuel Koller, Fabienne Maurer, Stéphanie Meier, Thierry Nouspikel, Javier Sanz, Christiane Zweier, Marc Abramowicz, Wolfgang Berger, Sven Cichon, André Schaller, Andrea Superti-Furga, Valérie Barbié, Anita Rauch

**Affiliations:** 1 Institute of Medical Genetics (IMG), University of Zurich (UZH), Wagistrasse 12, CH-8952 Zurich, Switzerland; dennis.kraemer@medgen.uzh.ch; 2 Swiss Institute of Bioinformatics (SIB), Clinical Bioinformatics, CH-1202 Geneva, Switzerland; dillenn.terumalai@sib.swiss (D.T.); valerie.barbie@sib.swiss (V.B.); 3 Swiss Institute of Bioinformatics (SIB), Swiss-Prot Group, CH-1211 Geneva, Switzerland; livia.famiglietti@sib.swiss; 4 Medical Genetics, Institute of Medical Genetics and Pathology, University Hospital Basel and University of Basel, CH-4031 Basel, Switzerland; isabel.filges@usb.ch (I.F.); pascalphilippe.joset@usb.ch (P.J.); stephanie.meier@usb.ch (S.M.); sven.cichon@usb.ch (S.C.); 5 Institute of Medical Molecular Genetics (IMMG), University of Zurich (UZH), Wagistrasse 12, CH-8952 Zurich, Switzerland; koller@medmolgen.uzh.ch (S.K.); berger@medmolgen.uzh.ch (W.B.); 6 Division of Genetic Medicine, Lausanne University Hospital (CHUV), CH-1011 Lausanne, Switzerland; fabienne.maurer@chuv.ch (F.M.); asuperti@unil.ch (A.S.-F.); 7 Genetic Medicine Division, Diagnostics Department/Center for Genomic Medicine, Geneva University Hospitals (HUG), 1206 Geneva, Switzerland; thierry.nouspikel@hcuge.ch (T.N.); marc.abramowicz@hcuge.ch (M.A.); 8 Department of Human Genetics, Inselspital, Bern University Hospital, CH-3010 Bern, Switzerland; javier.sanz@insel.ch (J.S.); christiane.zweier@insel.ch (C.Z.); andre.schaller@insel.ch (A.S.); 9 Neuroscience Center Zurich (ZNZ), University and ETH Zurich, CH-8057 Zurich, Switzerland; 10 Zurich Center for Integrative Human Physiology (ZIHP), University of Zurich, CH-8057 Zurich, Switzerland

**Keywords:** *SwissGenVar*, Switzerland, NGS, expert-curated variant interpretation, national mutation database, genotype–phenotype database, personalized medicine

## Abstract

Large-scale next-generation sequencing (NGS) germline testing is technically feasible today, but variant interpretation represents a major bottleneck in analysis workflows. This includes extensive variant prioritization, annotation, and time-consuming evidence curation. The scale of the interpretation problem is massive, and variants of uncertain significance (VUSs) are a challenge to personalized medicine. This challenge is further compounded by the complexity and heterogeneity of the standards used to describe genetic variants and the associated phenotypes when searching for relevant information to support clinical decision making. To address this, all five Swiss academic institutions for Medical Genetics joined forces with the Swiss Institute of Bioinformatics (SIB) to create *SwissGenVar* as a user-friendly nationwide repository and sharing platform for genetic variant data generated during routine diagnostic procedures and research sequencing projects. Its aim is to provide a protected environment for expert evidence sharing about individual variants to harmonize and upscale their significance interpretation at the clinical grade according to international standards. To corroborate the clinical assessment, the variant-related data will be combined with consented high-quality clinical information. Broader visibility will be achieved by interfacing with international databases, thus supporting global initiatives in personalized healthcare.

## 1. Introduction

The assessment of individual genetic risk factors and the classification of molecular diseases based on genetic contributions are hallmarks of personalized medicine [1,2,3,4,5]. Next to common genetic variants predisposing to or modulating common diseases, newer evidence also indicates a significant role of individually rare variants in frequently mutated genes with strong functional consequences [6,7]. Large-scale germline genetic testing is technically feasible today but is hampered by the difficulties in interpreting the clinical significance of variants, lack of knowledge about genotype–phenotype correlations, and long-term clinical history [8,9]. Accurate pathogenicity interpretations of genetic variants are crucial not only for appropriate medical decision making based on genetic evidence [10], but also for the correct stratification of research findings by genetic results [11]. A variety of international database initiatives aim to facilitate genetic variant assessment. However, these are often limited to specific genes and/or (types of) genetic diseases/alterations and contain insufficient, conflicting, or sometimes even incorrect public entries [12,13]. In most cases, they fail to provide accompanying valid clinical data for variant interpretation in their respective phenotypic contexts [14,15]. Moreover, as genetic variation commonly differs between ethnicities, international data collection may not be representative and comprehensive for specific populations. The importance of local and national genetics has been illustrated by the Genome of the Netherlands initiative [16]. Therefore, the next major challenge in personalized medicine will be the expansion of high-quality genotype–phenotype databases that provide “knowledge” over “data” to enable, but not dictate, accurate clinical care through rigorous quality management and a sustainable expert variant curation and classification process [17,18].

In Switzerland, research and diagnostic germline genetic testing is strictly regulated and subject to regular accreditation procedures. Accordingly, research involving germline genetic data and diagnostic genetic testing is mainly conducted by highly specialized centers, including universities and cooperating clinical centers for Medical/Human Genetics. The use of next-generation sequencing (NGS) technologies as the standard of care creates a rich source of genetic data with in-depth clinical variant assessments. These data are currently not being systematically collected, as there is no nationwide academic or public database of genetic variants obtained from diagnostic procedures or from sequencing research projects in Switzerland. Therefore, the considerable potential to promote the sharing of diagnostic-grade genomic data with patient-consented, high-quality clinical information remains largely untapped. Besides data protection issues, this may be explained, particularly for Swiss institutions, by the current lack of agreed harmonized standards and concepts for the collection and exchange of genetic data as well as the absence of an appropriate and secure repository infrastructure for genomic and related patient data.

To overcome these difficulties and leverage the high-quality genetic and associated clinical data generated at Swiss academic institutions, all five Swiss university centers for Medical/Human Genetics, together with the Swiss Institute of Bioinformatics (SIB, Clinical Bioinformatics), have made a national effort to create the *SwissGenVar* platform within the framework of an infrastructure development project of the Swiss Personalized Health Network (SPHN) [19] (project page available at [20]). *SwissGenVar* aims to create a protected, nationwide repository for germline variants identified in patients and research projects by Swiss clinical genetics laboratories, with accompanying high-quality clinical data and an efficient joint platform for harmonizing and up-scaling expert-curated variant interpretation. To this end, *SwissGenVar* ensures the interoperability with international databases and provides the methodological and technical prerequisites for national and international sharing/storage of genomic data and evidence for standardized variant pathogenicity assessment. This will considerably facilitate consensus variant classification by clinical genetics experts. Furthermore, *SwissGenVar* allows for the harvesting of patient-consented clinical data generated during routine healthcare to assess the clinical significance of a variant for a specific disease, in synopsis with the associated phenotypic features.

Within this project, we have therefore defined an interoperable set of genetic and non-identifying clinical data for variant data sharing/storage and clinical interpretation, a consistent process for uploading and annotation of genetic variant files, and a data ontology appropriate for the presented purposes of *SwissGenVar*. Accessibility for clinicians and researchers has been realized through an efficient, scalable, and user-friendly IT infrastructure, integrated into the secure BioMedIT [21] landscape of the SPHN. Currently, *SwissGenVar* is only accessible to the project partners, with the scope to expand to further academic and non-academic institutions to establish itself as the Swiss one-stop platform for interpreting germline genetic variants. Thus, *SwissGenVar* may substantially foster personalized healthcare as well as be a necessary first step towards the scale-up of clinical-grade genetic testing and data sharing in Switzerland.

## 2. Materials and Methods

### 2.1. Sensitive Data Hosting and Transfers

The *SwissGenVar* infrastructure development project has been initially funded by the Swiss Personalized Health Network (SPHN) [19] initiative, which builds on the Swiss national BioMedIT infrastructure, specifically implemented for hosting sensitive data. It therefore uses all the tools provided by these initiatives and follows their requirements.

The *SwissGenVar* application and data are hosted on the secure SENSA (Secure Sensitive Data Processing Platform) BioMedIT [21] node in Lausanne and comply with the SPHN and BioMedIT tools and the related Information Security Policy [22]. Data transfers are ensured by the SPHN SETT (Secure Encryption and Transfer Tool) data [23], which encrypts, securely transfers, and decrypts data.

Users’ identities and access are managed using a Keycloak [24] instance, which requires SWITCH eduID [25] two-factor authentication for login. Keycloak is an open-access IAM platform that secures web applications and RESTful web services using standard protocols such as OAuth2.0, OpenID Connect 1.0. In addition, access to the system is restricted to the whitelisted IP (Internet Protocol) address ranges of each participating institution.

All data used in the development of the platform and shown in the figures are for fictitious individuals, not real patients.

### 2.2. Software Development

*SwissGenVar* is a web-based application whose backend is written in PHP (using the Laravel framework) and relies on a PostgreSQL database. The frontend is based on Vue.js (using the Nuxt framework). The bioinformatics pipeline runs on a SLURM cluster.

### 2.3. Public Data Sources

For all variants in the VCF (Variant Call Format) files, some public information is automatically gathered by the *SwissGenVar* platform using a local instance of the Ensembl Variant Effect Predictor (VEP) [26] deployed on the SENSA BioMedIT node. This information currently includes the variant type and effect, the genomic position of the variant, and the HGVS (Human Genome Variation Society) variant nomenclature [27].

## 3. Results

### 3.1. SwissGenVar Governance and Layers of Access

**Figure 1:**
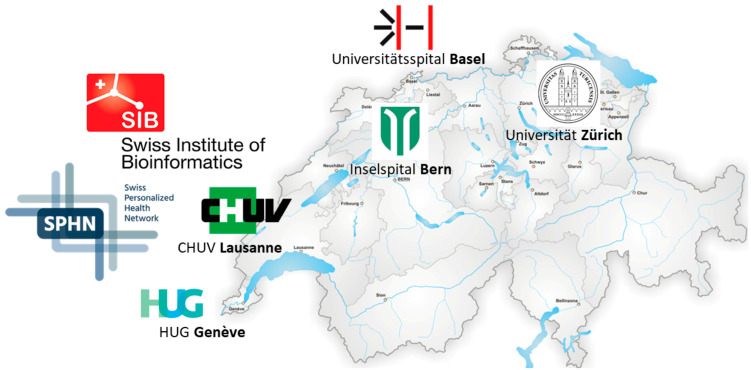
Institutions involved in the *SwissGenVar* consortium: University Hospital Basel, Medical Genetics; Department of Human Genetics, Inselspital, Bern University Hospital; Medical Genetics Service/Center for Genetic Medicine, Geneva University Hospital (HUG); Medical Genetics Service/Division of Genetic Medicine, Lausanne University Hospital (CHUV); Institute of Medical Genetics (IMG), University of Zurich (UZH); Institute of Medical Molecular Genetics (IMMG), University of Zurich (UZH); Swiss Institute of Bioinformatics (SIB); Swiss Personalized Health Network (SPHN). (Licence: Tschubby, Karte Schweiz, Institutions Involved in the SwissGenVar Consortium by Kraemer, Dennis et al., CC BY-SA 3.0).

For the implementation and administration of *SwissGenVar*, a multicenter consortium of all five academic centers for Medical Genetics in Switzerland and the Swiss Institute of Bioinformatics (SIB) (Figure 1) has been formed, which is governed by the Steering Board, as defined in the *SwissGenVar* Consortium Agreement. In order to combine the use for research and the highest level of data protection, the platform is composed of two different modules with different potential access layers, which are specified by the Data Transfer and Use Agreement (DTUA). The access-controlled instance is designed to share genetic data and non-identifying associated clinical/demographic metadata in view-only mode, including data submitted by any other registered group. Registered users belonging to a registered group may also modify their own data or metadata. Access to the data stored in the access-controlled instance is restricted to registered users of the consortium (full access layer). However, upon approval by the Steering Board, data from the access-controlled instance (including personal data) may be made accessible to users belonging to a third-party group if required for a specific research study and if approved by the competent ethics committee (restricted access layer). By contrast, the public instance is intended to make stand-alone variants and aggregated patient/proband data (without any information related to the specific patient/proband or sample) publicly available and will be freely accessible to all interested parties without registration (public access layer).

### 3.2. Standardized SwissGenVar Dataset Specifications

**Figure 2:**
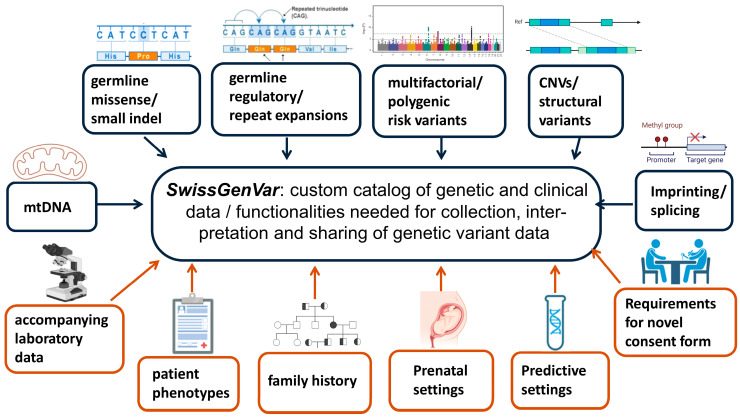
Synopsis of the clinical (*orange*) and laboratory (*grey*) working groups. These elaborate the minimal and extended datasets of genetic and clinical data, as well as the functionalities pertinent to the collection, sharing, and interpretation of genetic variants. At the operational level, a regularly meeting cross-expert team was installed for content implementation.

One of the key concepts of *SwissGenVar* is the combination of diagnostic-grade genetic variant-related data with accompanying consented high-quality basic clinical information to corroborate their diagnostic utility. To harmonize the variant-related and phenotypic ontologies, a cross-expert working group defined both a minimal and extended genetic and clinical dataset pertinent for data sharing/storage and the standardized interpretation of the clinical significance of genetic variants, which were approved by the *SwissGenVar* Board (Figure 2). After several rounds of in-depth discussions and board meetings, dedicated clinical and laboratory working groups, led by clinical experts in the respective field, elaborated a comprehensive and granular portfolio of parameters and functionalities needed for the objectives of *SwissGenVar* (Table 1).

To ensure interoperability with international databases and other SPHN projects, *SwissGenVar* follows established international standards and the SPHN guidelines for Interoperability Data Standard and Tool Collection [22] wherever applicable. For most items, well-defined existing ontologies are used. However, for those data fields relevant to the *SwissGenVar* project for which no appropriate data standard was available, the consortium had to define and adapt an internal data catalogue that reflected the consensus between the practices of the different partner institutions.

Furthermore, *SwissGenVar* allows for automated variant annotation from a variety of sources and implements direct links to the well-established NCBI ClinVar [28] and Single Nucleotide Polymorphism Database (dbSNP) [29], as well as to the Human Gene Mutation Database (HGMD) [30], DECIPHER [31], LOVD (Leiden Open Variation Database) [32], and SVIP-O [33], the latter being a Swiss SPHN platform for the clinical interpretation of genetic variants in oncology (Swiss Variant Interpretation Platform for Oncology). Additionally, the widely-used predictive algorithms SIFT (Sorting Intolerant From Tolerant) [34] and PolyPhen-2 [35] for the *in silico* assessment of amino acid substitutions are implemented using VEP.

### 3.3. Data Management and Application Workflow

**Figure 3:**
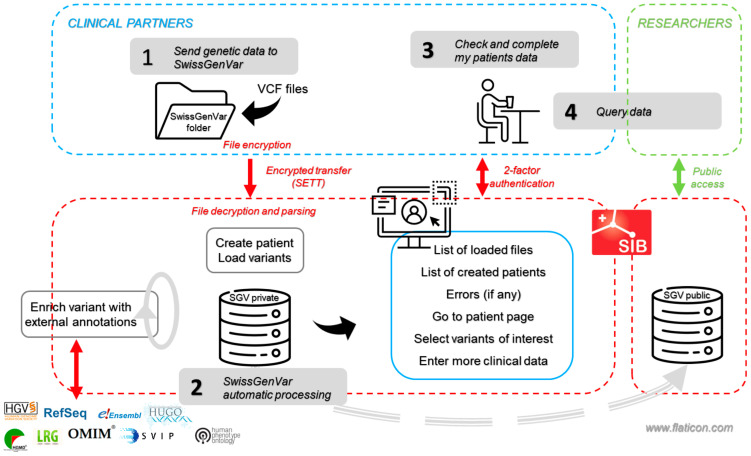
*SwissGenVar* application workflow. The *SwissGenVar* partners provide the genetic variant data to the *SwissGenVar* database in VCF format. The data are encrypted and transferred securely (*step 1*) using the SPHN Secure Encryption and Transfer Tool (SETT). Upon transfer to the *SwissGenVar* private/main application server, the files are decrypted and parsed to create patient entries and load the genetic variants into the platform. The variant entries are automatically enriched with selected external public annotations (*step 2*). At this stage, the partners can connect to their protected account using two-factor authentication to verify the transfer of their data files and start adding clinical information about their patients directly on the *SwissGenVar* interface (*step 3*). They can also query the entire database to go to specific patient pages and select variants of interest using multiple predefined filters (*step 4*). In a future step, *SwissGenVar* will also integrate a publicly accessible platform of aggregated variant-related and clinical information for personalized medicine research. (Symbolic figures were partly created using BioRender.com).

The project partners provide high-quality genetic data, mostly from NGS procedures (exome and genome sequencing or other methods in the form of VCF files [Variant Call Format]), either derived from research studies or from diagnostic tests with general or dedicated *SwissGenVar* consent, which are complemented by a minimal set of basic clinical information from the patient’s relevant medical history (Figure 3). These genetic and clinical data are generated either directly by the participating laboratories or by the hospital’s Clinical Data Warehouses (CDW), depending on the setup of each partner institution. In both cases, the genetic data are encrypted and securely transferred using the SPHN BioMedIT transfer tool [23] and are stored and accessed according to BioMedIT access and security standards [24,25]. Subsequently, after the decryption and parsing of the transferred files, patient entries are created, and variant calls from the VCF files are loaded onto the platform. Before being loaded, the genetic data undergo a technical basic check to ensure compliance with the requested VCF file format. The user can then select individual variants as being “of interest”, so that they are displayed in priority on the interface.

Additionally, using a local instance of the Ensembl Variant Effect Predictor (VEP), *SwissGenVar* automatically retrieves publicly available annotations for each variant, such as the gnomAD (Genome Aggregation Database) population frequency, variant effect, and the presence of the variant in public databases such as NCBI ClinVar. The implementation of additional public annotations by integrating APIs from further data sources is being investigated. For the patient phenotypic features, *SwissGenVar* allows clinical experts to manually enter clinical information and specific findings relevant to the variant assessment on their patients via its web interface, using standardized vocabularies agreed upon during the project. Only the data providers are allowed to modify their own data in case of corrections or the addition of clinical data.

### 3.4. SwissGenVar Database Structure, Data Query, and Data Display

**Figure 4:**
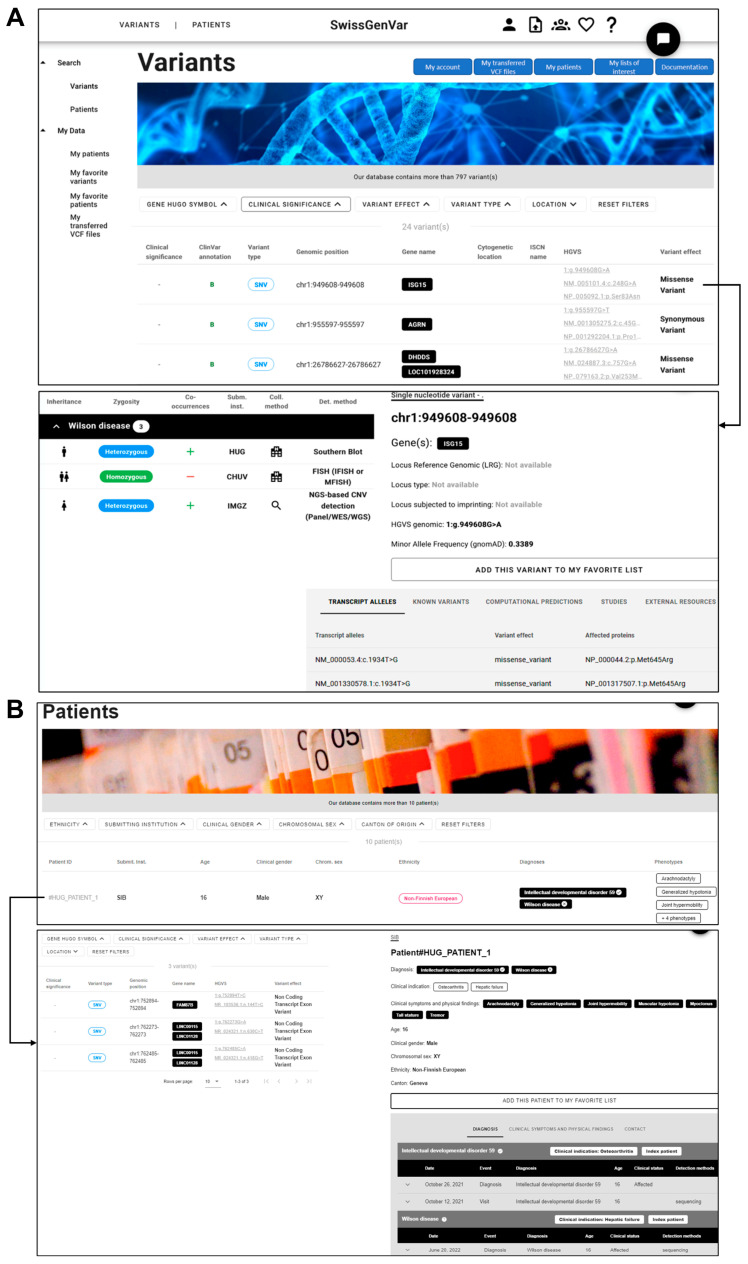

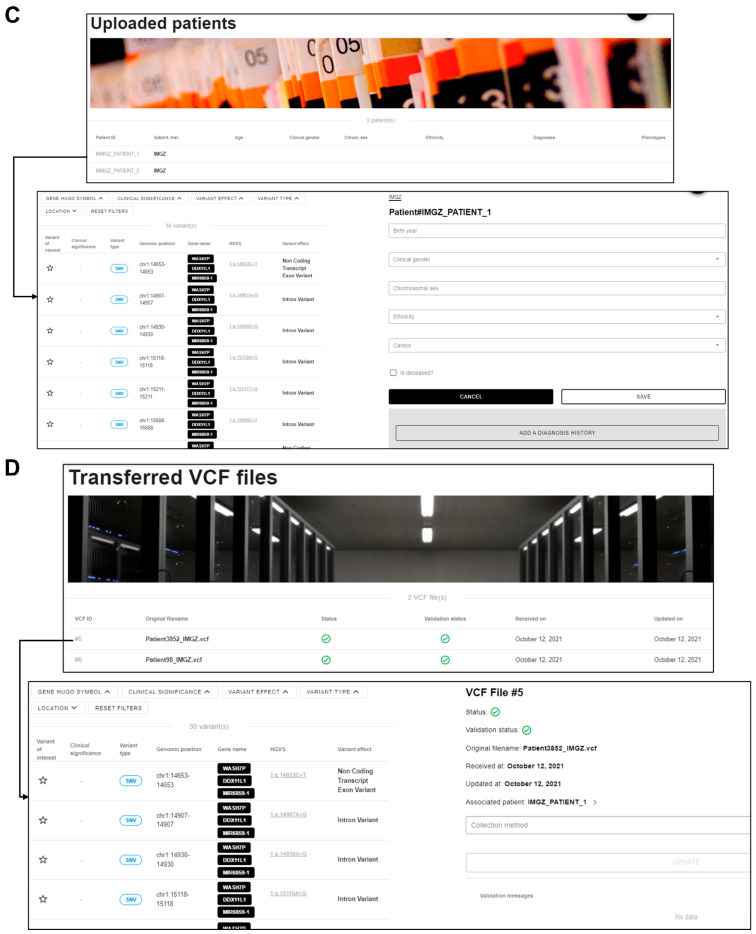
*SwissGenVar* variant (**A**) and patient (**B**) query pages. These pages consist of the filter bar (*1*); table of all/matching variants/patients with selected annotations (*2*); search panel to switch between variant and patient query pages (*3*); selection panel “My data” (*4*) to view and edit the patients submitted by the user (under the overview “Uploaded patients” and the detailed page (**C**)), respectively) to view the personal lists of the variants/patients of interest or to view and edit the VCF files transferred by the user (under the overview “Transferred VCF files” and the detailed page (**D**), respectively); menu bar (*5*) with a personal account and link to the VCF upload service. By clicking on a row of the respective table, the user is redirected to the individual detailed page with further information, and in the case of the “My patients” and “Transferred VCF files” pages, to edit his/her own patients and VCF files. It should be noted that all data presented are for fictitious individuals, not real patients.

We developed a graphical user interface to visualize and query the data, enabling the users to explore genetic variants in a gene and/or patient of interest or to retrieve patients with specific phenotypic features. Queries can be issued either from a variant or a patient query page (Figure 4). This interface allows for the creation of a custom query based on the user’s interest, with one or multiple criterion filters to search the database and display all the variants or patients matching the selected filtering criteria. The query result is displayed in a variant or patient results table, respectively, showing only the selected information items that can be compared or searched for. However, once a specific variant or patient has been selected by clicking on the corresponding row of a results table, the user can access the individual detailed page providing more granular information about the variant or the patient of interest. Thus, the detailed variant page includes a table of all the patients harboring that specific variant along with selected related information, as well as automatically retrieved variant annotations, as described in Table 1. The detailed patient page contains a table of variants detected in the patient of interest (obtained from the VCF files) and provides various phenotypic features. If no filter is used, the variant and patient tables list all the variants of interest by default and patients present in the database. The “Uploaded patients” and “Transferred VCF files” pages, which are accessible via the “My Data” selection panel or menu at the top of the interface, assist users in managing their own data and provide an overview of their submitted patients and transferred VCF files, including their (validation) status. On the individual detailed patient page, the clinical partner of the submitting institution can complement the patient entry with a standardized dataset of non-identifying clinical and demographic information and add the granular history of medical contacts with the clinical/phenotypic findings obtained and potential genetic diagnoses. In addition, the data provider can prioritize clinically (potentially) significant variants by flagging them as “of interest” (by clicking on the asterisk icon on the left side of the variant table), which can likewise be completed directly on the detailed page of the respective submitted VCF file. Finally, the application offers the possibility to add variants and patients to the user’s favorites list under the individual detailed page. A notification system will be established to inform the users of any changes or updates to their variants or patients of interest.

**Table 1:** Standardized core dataset. (**A**) Established data catalogues and data sources used in *SwissGenVar*; (**B**) Internal data catalogues defined for *SwissGenVar*.

(**A**)				
**Information**	**Data Source**	**Obtained by**	**Full Name**	**Description**
Clinical indication	HPO [36]	Manual entry	Human Phenotype Ontology	Key phenotype, leading to genetic evaluation selected from standardized vocabulary of phenotypic abnormalities encountered in human disease
ClinVar clinical significance	ClinVar [28]	Variant Effect Predictor (VEP)	ClinVar	Public archive of reports of the relationships among human variations and phenotypes, with supporting evidence
Clinical significance	ACMG [37]	Manual entry	American College of Medical Genetics	ACMG five-tiered classification system for variants: pathogenic, likely pathogenic, uncertain significance, likely benign, benign
Diagnosis	OMIM [38]	Manual entry	Online Mendelian Inheritance in Man	Monogenic etiologic diagnosis
Ethnicity (self-reported)	gnomAD [39] categories	Manual entry	Genome Aggregation Database	gnomAD populations: African/African American, Amish, Latino/Admixed American, Ashkenazi Jewish, East Asian, Finnish, Non-Finnish European, Middle Eastern, South Asian, other
Frequency	gnomAD	VEP	Genome Aggregation Database	gnomAD global minor allele frequency (MAF)
Gene name	HGNC [40]	VEP	Human Genome Organisation Gene Nomenclature Committee	Unique gene name according to the HUGO gene nomenclature
Inheritance of the disease	OMIM categories	Manual entry	Online Mendelian Inheritance in Man	OMIM categories: AD—autosomal dominant, AR—autosomal recessive, PD—pseudoautosomal dominant, PR—pseudoautosomal recessive, DD—digenic dominant, DR—digenic recessive, IC—isolated cases, ICB—inherited chromosomal imbalance, Mi—mitochondrial, Mu—multifactorial, SMo—somatic mosaicism, SMu—somatic mutation, XL—X-linked, XLD—X-linked dominant, XLR—X-linked recessive, YL—Y-linked
Inheritance of the variant	Following DECIPHER [31] categories	Manual entry	Database of genomic variation and phenotype in humans using Ensembl Resources	Following DECIPHER categories: de novo constitutive; de novo mosaic; paternally inherited, constitutive in father; paternally inherited, mosaic in father; maternally inherited, constitutive in mother; maternally inherited, mosaic in mother; biparental; imbalance arising from a balanced parental rearrangement; inherited mosaic; unknown
Phenotype	HPO	Manual entry	Human Phenotype Ontology	Detailed clinical features selected from standardized vocabulary of phenotypic abnormalities encountered in human disease
Transcripts	RefSeq [41], Ensembl [42]	VEP	NCBI Reference Sequence Database; Ensembl	RefSeq: a comprehensive, integrated, non-redundant, well-annotated set of reference sequences, including genomic DNA, transcripts, and proteins; Ensembl: a genome browser for vertebrate genomes that supports research in comparative genomics, evolution, sequence variation, and transcriptional regulation
Variant description	HGVS [27]	VEP	Human Genome Variation Society	This nomenclature is used for the description of sequence variants (namely HGVSg, HGVSc, and HGVSp)
(**B**)				
**Information**	**Possible Values**			**Remark**
Age at onset	−1 (prenatal), 0, 0.1, 0.2, …, 100			Range of numbers for the age of onset in years
Aneuploidies	Yes; no			
Canton	List of Swiss cantons, plus “non-Swiss”			
Causality	Causative; likely causative; probably not causative; not causative; VUS; variant in a GUS			Causality following clinical judgement
Chromosomal sex	XX; XY; other			
Clinical gender	Male, female, ambiguous, transgender			
Karyotypic sex	45X, 46XX, 46XY, 47XXY, 47XYY, 47XXX (intended as expandable list)			Content is conditional on the value of “other” in “Chromosomal sex”
Clinical status	Affected; partially affected; potentially affected; not affected			Defined fields/filters: “clinical status change to”; “clinical status at last clinical assessment”
Co-occurrences	Yes; no			Co-occurrence of more than one causative variant
Collection method	Case-control; clinical testing; reference population; research; other; unknown			
Cytogenetic location	The cytogenetic location of the variant displayed as CHROM_NUMBERq/pCYTOGENETIC_BAND			
Detection method	Sequencing; fragment analysis; Southern Blot; conventional cytogenetics; FISH (IFISH or MFISH); Array (Oligo or SNP); qRT-PCR; MLPA; NGS-based CNV detection (Panel/WES/WGS); other; not performed			
Gene locus type	Protein-coding gene; non-coding RNA gene; long non-coding RNA; microRNA; ribosomal RNA; transfer RNA; small nuclear RNA; small nucleolar RNA; other; locus subjected to imprinting			Partly coming from HUGO Gene Nomenclature Committee (HGNC) [40]
Index patient	Yes; no			
Location	Genomic position			GRCh37 as genome reference build [43]
Locus subjected to imprinting	Yes; no; unknown			
Patient identifier (ID)	The patient ID refers to an internal *SwissGenVar* specific unique identifier that is generated when the patient is created in the system			Patient/sample ID of the submitting institution is recorded as well
Submitting institution	One acronym per partner institution			
Variant effect	Missense variant; nonsense variant; splice region variant; splice acceptor variant; splice donor variant; regulatory region variant; promoter region variant; inframe insertion; inframe deletion; intron variant; synonymous variant; stop lost variant; start lost variant; frameshift variant; upstream gene variant; downstream gene variant; intergenic variant; non-coding transcript exon variant; TF binding site variant; 5′ UTR variant; 3′ UTR variant; exon deletion; exon duplication; contiguous gene deletion; contiguous gene duplication			Adapted to Sequence Ontology (SO) [44] terms
Variant location	Coding region; splicing region; 5′ UTR; 3′ UTR; upstream gene; downstream gene; promoter region; intronic region; regulatory region; intergenic region			
Variant type	CNV—amplification; CNV—deletion; CNV—insertion/duplication; complex rearrangement; conversion; deletion; deletion–insertion; duplication; insertion; methylation/epigenetic change; repeat variation; structural variant; substitution			
Variant zygosity	Heterozygous; homozygous; hemizygous; mitochondrial heteroplasmy; mitochondrial homoplasmy; unknown; mosaic; chimeric; ambiguous			

CNV, copy number variation; FISH, fluorescence in situ hybridization (IFISH, interphase-FISH; MFISH, metaphase-FISH); GUS, gene of uncertain significance; MLPA, multiplex ligation-dependent probe amplification; qRT-PCR, quantitative reverse transcription PCR (polymerase chain reaction); SNP, single nucleotide polymorphism; TF, transcription factor; WES, whole-exome sequencing; WGS, whole-genome sequencing; VUS, variant of unclear significance; UTR, untranslated region.

## 4. Discussion

*SwissGenVar* aims to use datasets with general consent or with dedicated *SwissGenVar* consent to evaluate the landscape of (clinically relevant) genetic variants in Switzerland to improve variant interpretation and risk assessment by studying genotype–phenotype correlations and the natural history of genetic predispositions and disorders. This shall increase our knowledge and result in appropriate standard operating procedures and structures for improved patient care. Therefore, *SwissGenVar* intends to be a national repository for genetic findings from available and consented genetic datasets across all five academic Medical Genetic institutions in Switzerland as well as jointly assess their clinical significance to implement standard operating procedures and improved genetic diagnostics and patient care. SwissGenVar’s main achievement *is to enable* the collection and sharing of genetic and associated clinical data via secure data transfer and access/retrieval by the project partners. At the same time, it provides a platform for knowledge sharing about variant-related evidence to harmonize and upscale their significance interpretation at the clinical grade, with interoperability with international efforts.

For this purpose, *SwissGenVar* supports granular multifactorial filtering for variants and patients in separate query interfaces and details “in-house” variant-related and clinical evidence such as data from local mutation and clinical databases, as well as segregation and experimental analyses. Additionally, *SwissGenVar* allows its users to submit published information, such as published literature reports and functional studies, and includes publicly searchable variant annotations and links to well-established variant databases following international standards. In addition to the collection of genetic variants found in Swiss subpopulations, the integration of the complete set of variant calls from the transferred VCF files, the granular history of medical contacts, and the portfolio of phenotypic findings can be considered as a major advantage over existing genotype-phenotype/variant databases. [45,46]. This allows for a comprehensive clinical assessment of variants in the synopsis of co-occurring candidate variants and the respective clinical features of the variant-carrying individual, which is supported by the option to flag several variants as “of interest” in the corresponding VCF files. To encourage expert discussions on the significance interpretation, a notification system will inform users of any changes or updates to the classification of individual variants or patients of interest. Finally, the *SwissGenVar* project has strongly contributed to the harmonization of diagnostic practices among the participating institutions by defining and standardizing ontologies for variant and related clinical data. The ontology catalogue has been made available to the SPHN Data Coordination Center (DCC) [47] to serve as a basis for other (and follow-up) projects in medical genetics.

Individual findings may be followed up, and depending on the consent provided, clearly pathogenic findings with high predictive value may be fed back to the referring medical geneticist for genetic counselling of the patient. The knowledge gained for individual variants shall be annotated in the *SwissGenVar* database and may become publicly accessible in a public outlet of the platform, integrating interfaces to international database efforts. So far, the platform is only available to the partner groups, with the scope for expansion to other academic and non-academic institutions.

## 5. Conclusions

In conclusion, *SwissGenVar* provides a protected platform for the nationwide collection of germline genetic variants and the sharing of associated evidence and curated variant significance interpretations by clinical genetics experts, integrating a consistent genetic variant file upload and a semi-automated annotation/curation pipeline. As such, *SwissGenVar* may be considered as a necessary first step towards harmonizing and scaling-up clinical-grade genetic testing in Switzerland, thereby fostering personalized health research involving genetic risk stratification and disease classification.

## Data Availability

The *SwissGenVar* documentation page and public project page are available at https://pages.sib.swiss/project/swissgenvar-doc/ and https://sphn.ch/network/projects-old/infrastructure-development-projects/project-page-swissgenvar/, respectively. The project GitLab site is available at the URL https://gitlab.sib.swiss/clinbio/swissgenvar/sgv-knowledge/-/tree/master.
